# Media Representation of the Ethical Issues Pertaining to Brain–Computer Interface (BCI) Technology

**DOI:** 10.3390/brainsci14121255

**Published:** 2024-12-14

**Authors:** Savannah Beck, Yuliya Liberman, Veljko Dubljević

**Affiliations:** 1 College of Humanities and Social Sciences, North Carolina State University, Raleigh, NC 27695, USA; sbeck4@ncsu.edu; 2 College of Liberal Arts, Temple University, Philadelphia, PA 19122, USA; yuliya.liberman@temple.edu

**Keywords:** brain–computer interface (BCI), ethics, media, public discourse

## Abstract

Background/Objectives: Brain–computer interfaces (BCIs) are a rapidly developing technology that captures and transmits brain signals to external sources, allowing the user control of devices such as prosthetics. BCI technology offers the potential to restore physical capabilities in the body and change how we interact and communicate with computers and each other. While BCI technology has existed for decades, recent developments have caused the technology to generate a host of ethical issues and discussions in both academic and public circles. Given that media representation has the potential to shape public perception and policy, it is necessary to evaluate the space that these issues take in public discourse. Methods: We conducted a rapid review of media articles in English discussing ethical issues of BCI technology from 2013 to 2024 as indexed by LexisNexis. Our searches yielded 675 articles, with a final sample containing 182 articles. We assessed the themes of the articles and coded them based on the ethical issues discussed, ethical frameworks, recommendations, tone, and application of technology. Results: Our results showed a marked rise in interest in media articles over time, signaling an increased focus on this topic. The majority of articles adopted a balanced or neutral tone when discussing BCIs and focused on ethical issues regarding privacy, autonomy, and regulation. Conclusions: Current discussion of ethical issues reflects growing news coverage of companies such as Neuralink, and reveals a mounting distrust of BCI technology. The growing recognition of ethical considerations in BCI highlights the importance of ethical discourse in shaping the future of the field.

## 1. Introduction

The advent of medicine and medical technology has had an immeasurable impact on human history [1]. Medical technology has the potential to cure diseases that may have once been a death sentence, take away debilitating pain, and lengthen our lifespans significantly. This power of medical technology also came with the recognition that careful scrutiny, especially in terms of safety and effectiveness, is needed. On the other hand, consumer technologies are held to much lower standards and can be marketed even if there is no proof that they are effective. Brain–computer interfaces (BCIs) are rapidly gaining traction in the public consciousness as a promising new technology that straddles both medical and consumer realms. BCIs capture brain signals and transmit them to external sources, allowing the user mental control over devices such as prosthetics [2]. Although public discourse over BCI technology is fairly recent, BCI research began with animal trials in 1969 before moving on to human trials in the 1990s [3]. BCIs were often heralded as a way to return bodily function and autonomy to disabled patients, such as those suffering from locked-in syndrome or those with missing limbs. However, as this technology has progressed, it is now being considered for personal, professional, and even military applications [4].

**Figure 1:**
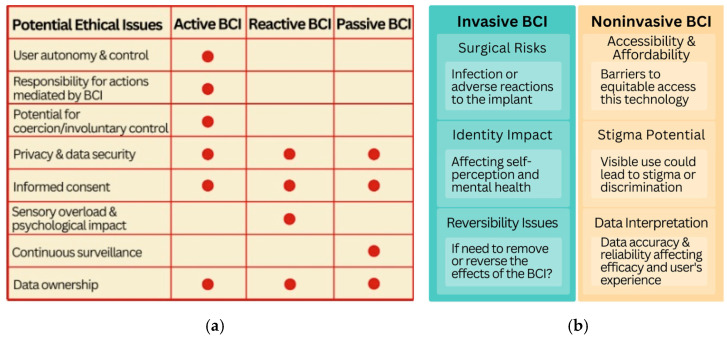
(**a**) BCI Ethical Issues based on User Interaction; (**b**) BCI Ethical Issues based on Invasiveness (cf. [2,9]).

BCIs function by decoding neural information and using it to communicate with external devices [5]. Most notably, the BCIs that are currently being developed and have garnered attention are the systems of Cyberkinetics, NeuroPortTM, Brain Gate, and the Stentrode implants by the company Synchron and Neural Lace by the company Neuralink [6], each creating a unique way to use cognition to influence the commands of a machine or computer. These technologies aim to allow both individuals who are paralyzed in some capacity or are unable to speak to reign these abilities for speaking, walking, or manipulating objects [7,8], as well as offering a novel way to communicate with the technology we use every day for individuals without impairments such as by sending commands to a computer through brain signaling rather than through speech or manual engagement. There is a further distinction between passive and active BCI devices. Passive BCIs do not require action from the user and the device is able to read brain signals without any conscious input. Active BCIs refer to devices where the user actively modulates their brain waves in order to control the device or the input that the device receives. Invasive BCIs refer to devices that are implanted in the brain whereas non-invasive BCIs refer to devices that read brain signals using surface sensors that do not require direct contact with the brain. The degree of invasiveness and distinction of active versus passive BCI technology significantly influences the relevant ethical considerations and medical risks associated with the device discussed (see Figure 1).

With the broadening scope of application has come a more comprehensive range of ethical issues regarding the prevalence of BCIs. Concerns about issues such as privacy, inequality, and accessibility will only grow stronger as the technology continues to advance and could potentially be pushed into mainstream usage. Companies like Synchron [10], Paradromics [11], and Neuralink [12] have already been approved for human trials of invasive BCI devices by the FDA, and though their approval rests on the companies providing evidence that their devices must have a demonstrable medical use, there has already been discussion about the potential they have for “human enhancement” [13]. In the non-invasive, or wearable, BCI arena, multiple start-up companies compete to corner the wellness and cognitive enhancement device market [14]. However, the most striking example of such “better than well” strategies, Neuralink’s mission statement is to “create a generalized brain interface to restore autonomy to those with unmet medical needs today and unlock human potential tomorrow” [15]. Neuralink announced its human trial registry in September of 2023, and hosted a livestream showing the results of its first successful implant in March of 2024 on the social media platform “X”, previously known as “Twitter” [16]. As of the time of writing this paper, the livestream currently has 20 million views.

Due to the rise of social media for rapid information dissemination, scientific progress that may once have been restricted to academic articles and subject to a rigorous review process can now be spread through social media platforms [17]. Oftentimes, media can serve as a boundary between frequently inaccessible academic studies and the deliberate spread of misinformation. As previous studies on media representation of other emerging technologies have shown, the framing of information in the media can often influence public perception [18,19,20]. These shifts in public opinion can have far-reaching effects, such as influencing how future BCI research is conducted and how the devices are implemented and regulated. Since human trials for BCI technology are still in their infancy and have only recently garnered public attention, the initial discussion regarding ethical issues pertaining to the devices can have a profound impact on how BCI technology continues to advance [2].

Rapid review methodology [21] is ideal for time-limited assessment of a body of literature and for quickly informing the academic community and the public at large about the themes and salient direction. As such, our rapid review seeks to clarify the current media perception of BCIs by analyzing media articles that discuss ethical issues posed by the devices. Our study looks at articles from 2013 onwards to provide a perspective on how media representation has evolved with the emergence of modern-day BCIs, and where current media opinion stands. We seek to critically examine the most prominent ethical concerns and recommendations for future directions regarding BCIs, while also providing an analysis of the ethical frameworks and general tone used during the discussions. More specifically, we seek to dispel myths by identifying possible misinformation and fostering a comprehensive assessment of ethical implications. Also, we hope to inform current decision-making by providing nuanced information on the ethical debates surrounding different types of BCIs to policymakers, healthcare providers, developers, and the general public.

## 2. Materials and Methods

Our study used qualitative methods to evaluate how ethical issues regarding BCIs are portrayed to the public. For this purpose, we performed a database search on print media articles published from 2013 to 2024. We selected the year 2013 as the beginning of our study because this was the first year that had a substantial number of articles that matched our criteria, and because we wanted to gain a clear picture of the emerging public perception of BCI ethics in the last decade. Our initial search was conducted using LexisNexis on 21 February 2024, using the search terms “brain–computer interface” AND “ethic*”. LexisNexis is a comprehensive research platform that we used as a tool for searching for and accessing reliable media articles. Articles were filtered to include only those written in English. Since this review focuses on the general public’s perceptions of BCIs, content was filtered to include “web-based publications”, “newswires and press”, “industry trade press”, “magazines and journals”, “blogs”, “weblinks”, and “news transcripts”.

The search yielded a sample of 512 articles. After excluding duplicates, we were left with 434 articles. From this, we applied our exclusion criteria to filter out articles that grouped BCIs with other technology when discussing ethical issues, articles focused on technical descriptions of BCIs with only tangential mention of ethical issues, and TV or radio transcripts. These criteria excluded an additional 281 articles, leaving us with a final sample size of 153 articles. In order to have a better understanding of the more recent developments in reporting, we updated the search with data until the end of June 2024. The sample size of this search was 163 articles. After excluding duplicates, 130 articles remained. We then applied the same exclusion criteria, which yielded 29 included articles, with 134 articles excluded from the updated 2024 sample size. In summary, our searches yielded 675 articles in total. After iteratively applying our exclusion criteria, we were left with a final sample of 182 articles.

To establish intercoder reliability, we generated a pilot sample consisting of the first 10% of articles from each year. Two authors (SB and JL) independently coded the articles, and all discrepancies were settled by consulting a third coder (VD). We repeated this process until all unclear cases were resolved, and our final calculated intercoder reliability was 90.7%. Our coding scheme was discussed beforehand and refined by comparing our coding methods during the pilot sample. Namely, data were coded based on the abductive inference approach to qualitative research [22], which combines the strengths of deductive (top down) and inductive (bottom up) thematic analysis. Our coding consisted of establishing themes based on prior results in the reviews of the academic literature (e.g., [2]) and open coding for newer emerging themes. Following prior work [18], the coding structure was divided into the following five categories: (1) ethical issues, (2) ethical frameworks, (3) recommendation, (4) tone, and (5) application of technology. From these categories, we established several subcategories to additionally represent and chart the content of the articles.

Ethical Issues: This category represents the type of ethical issues discussed in the articles. This broad theme contains the subcategories Biohacking, Autonomy, Coercion/Consent, Privacy, Weaponization, Safety, Animal Welfare, Responsibility, Personhood/Identity, Accessibility, Inequality, Overpromising, Exploitation and Regulation. The subcategory “Biohacking” addressed concerns regarding the security of the device and the manipulation of people or data as a result. “Autonomy” coded for the right of the user to self-govern their body and mind. “Coercion/Consent” coded for the ability to freely choose to use the device. Articles discussing “Privacy” were coded for data concerns involving the companies that had provided the technology. “Weaponization” coded for ethical concerns about military use. Articles that mentioned “Safety” discussed the physical security of the device, whereas “Animal Welfare” specifically referred to animal safety, wellbeing and testing concerns. “Responsibility” included discussions on whether accountability for potential harm would fall on the device or the user. “Personhood” coded for general concerns about the loss of humanity to the device. Issues regarding “Accessibility” discussed the ability to acquire and use the device. “Inequality” discussed concerns of inequalities created or exacerbated by the usage of the device. “Overpromising” discussed the potential emotional harm due to advertising the devices as more capable than they are. “Exploitation” coded for the potential for researchers and stakeholders to exploit those with conditions that would benefit from BCI usage for profit. Finally, “Regulation” involved discussions of government involvement and policy issues.

Ethical Frameworks: This category discusses the articulation of ethical issues based on recognized applied ethics principles, such as autonomy, justice, non-maleficence, and beneficence, as they fit into broader ethical theories such as care ethics, deontology, and utilitarianism/consequentialism. The subcategory “Consequentialism” coded for articles that considered the ethical implications of BCI technology through the lens of whichever action resulted in the greatest good for society, as exemplified in the works of Bentham [23]. “Care ethics” coded for articles that focused on the moral worth of care and benevolence in interpersonal relationships [24]. The subcategory “Categorical Imperative” coded for articles that cautioned against using individuals as a means for an end using the ethical lens of deontological ethics, exemplified in the writings of Immanuel Kant [25]. The subcategory “Other” exists to quantify pluralistic ethical concerns, including common principles of biomedical ethics research [26] that do not fall into the above categories and are not prevalent enough to require a category of their own.

Recommendation: This category discusses the recommendations regarding the ethics of BCIs that were outright stated or implied by either the author or individuals quoted in the article. The “Encouraging Public Involvement” subcategory coded for the need for public participation and further dissemination of information regarding BCIs. “Avoiding Undesired Results” involved discussion of unintended side effects from the distribution of the devices. Recommendations of “Maintaining Choice” cautioned against social pressure or non-consensual usage of the device. “Maintaining Autonomy” coded for recommendations to prevent the loss of autonomy to the device. “Not For Public Use” involved discussions of restricting BCI usage to the medical field, while “Not For Sale/Profit” discussed concerns involving monetization of the devices. “Regulation” coded for suggestions of policy or government intervention. “Medical Oversight” referred to recommendations that medical practitioners continue to work closely with applications of BCIs. Finally, “More Research” coded for the general need for more research before the devices are made available for broader use.

Tone: This category coded for the general attitude that the articles took on while discussing BCI technology and was divided into “Positive/Enthusiastic”, “Negative/Critical”, and “Balanced/Neutral” subcategories. “Positive/Enthusiastic” included articles that predominantly focused on the benefits and practical potential of the devices. “Negative/Critical” included articles that predominantly focused on the risks or potential harm of the devices. Finally, “Balanced/Neutral” included articles that either took a neutral stance on BCIs or represented both positive and negative views of the devices in equal amounts.

Application: This category coded for the potential applications of BCI technology, as discussed in the articles. This included the subcategories “Medical” for usage in medicine in order to restore lost or absent function of the body, “Personal” for personal usage of the devices, “Professional” for usage as suggested or required by an employer or profession, and “Military” for usage of the devices by a military force.

## 3. Results

### 3.1. Year of Publication

**Figure 2:**
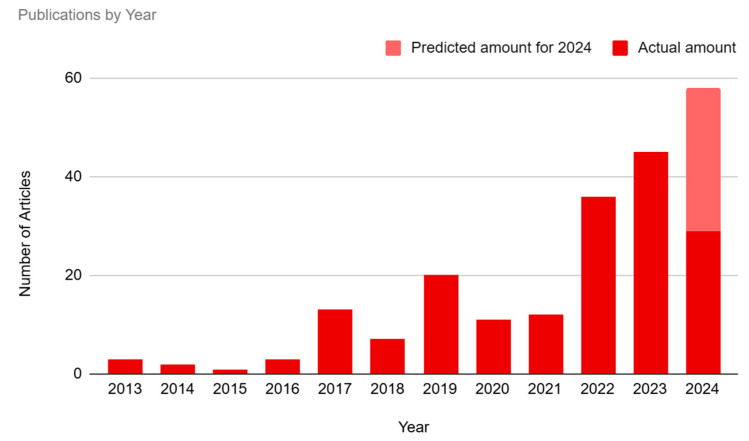
The number of articles per year. Predicted amount for the second half of 2024 was based on the actual amount of articles from the first half of 2024.

Our study showed an overall increase in media interest in BCI technology over time (see Figure 2 and Table S1 in Supplementary Materials). The number of articles published in 2013–2016 represented only 4.95% (n = 9) of the sample. There was a small spike in interest in the year 2017 (7.14%, n = 13), followed by a dip in 2018 (3.85%, n = 7) and then a subsequent spike in 2019 (10.99%, n = 20). Interest dipped again during 2020 (6.04%, n = 11) and 2021 (6.59%, n = 12), before hitting a peak in 2022–2024. The year 2022 represented 19.78% (n = 36) of the sample, and 2023 represented 24.73% (n = 45) of the sample. Our study has only evaluated the first half of 2024, which represents 15.93% (n = 29) of the articles. If current trends in 2024 continue throughout the rest of the year, a reasonable estimate, based on doubling the half-year amount, is that this will be the year with the highest media interest in BCI technology thus far.

### 3.2. Ethical Issues

**Figure 3:**
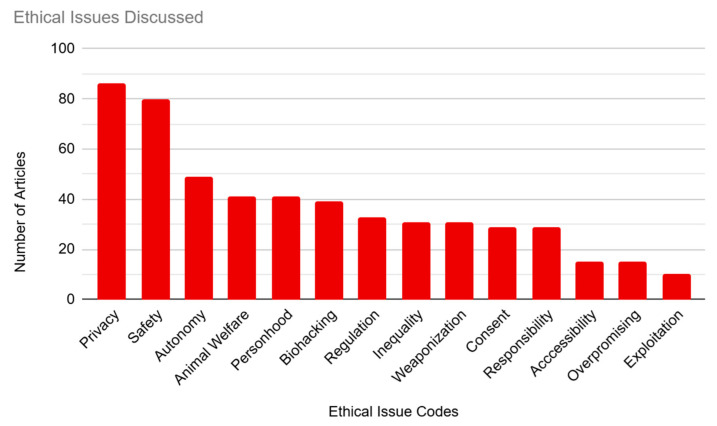
Ethical Issues Discussed in the Media.

The articles in our sample were coded based on the ethical issues they discussed in relation to BCIs (see Figure 3 and Table 1). This was our largest category, with 14 individual codes included. Most of our articles were coded for multiple ethical issues, with only 9.89% (n = 18) of articles coding for only one issue.

The most common ethical concern that we coded for was privacy, which was mentioned in 47.25% (n = 86) of the sample. This was followed by safety, at 43.96% (n = 80) of the articles. Concerns about autonomy were represented in 26.92% (n = 49) of the sample. Both “Animal Welfare” and “Personhood” tied at 22.53% (n = 41) of the sample. “Biohacking” was coded for in 21.43% (n = 39) of the sample. “Regulation” was coded for in 18.13% (n = 33) of the articles. The next few articles were involved in ties for their respective place. “Inequality” and “Representation” both represented 17.03% (n = 31) of the sample. “Coercion/Consent” and “Responsibility” each represented 15.93% (n = 29) of the sample. The subcategories “Accessibility” and “Overpromising” were each represented in 8.24% (n = 15) of articles. Finally, “Exploitation” was the least represented ethical concern, being mentioned in only 5.49% (n = 10) of the sample.

### 3.3. Ethical Frameworks

**Figure 4:**
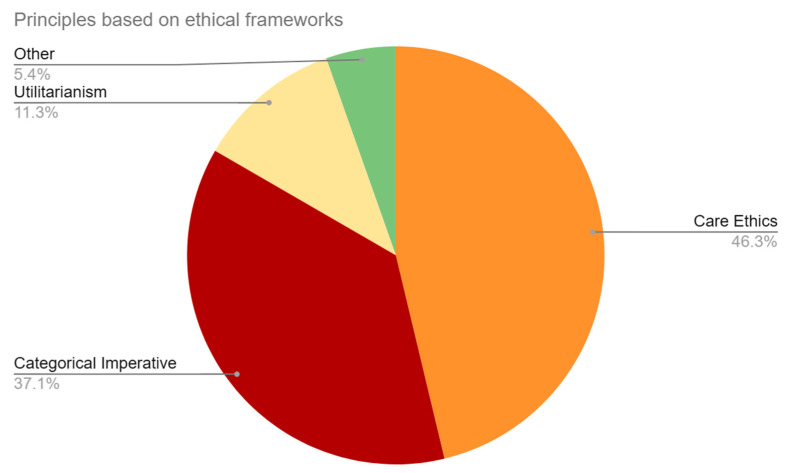
Ethical frameworks. This figure represents the percentage of each code mentioned out of the total number of ethical framework code mentions, as opposed to the percentage of each code out of the total number of articles as seen in Section 3.3.

This category coded for implied or outright stated ethical frameworks that were used to analyze the effects of BCI technology (see Figure 4). The majority of our sample applied the framework of care ethics in their discussion, or 60.99% (n = 111) of the articles. This was followed by the categorical imperative, which was used in 48.90% (n = 89) of the sample. The principle of utilitarianism was applied in 14.84% (n = 27) of the sample. Finally, 7.14% (n = 13) of the articles mentioned various pluralistic ethical principles.

### 3.4. Article Recommendation

**Figure 5:**
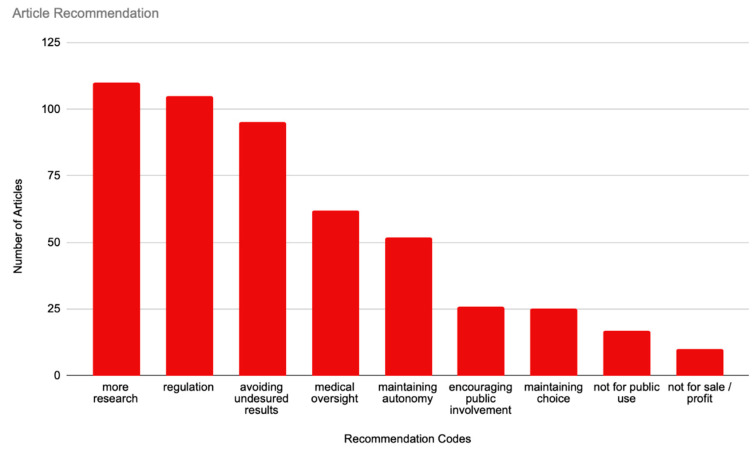
Recommendations.

This category coded for implicit or explicit recommendations by the authors or individuals quoted in the articles (see Figure 5). Our most prominent subcategory was “More Research”, which was coded in 60.44% (n = 110) of the articles. This was closely followed by “Regulation”, which was coded in 57.69% (n = 105) of the articles. “Avoiding Undesired Results” was represented in 52.20% (n = 95) of the sample. This was followed by “Medical Oversight”, which was recommended in 34.07% (n = 62) of the sample. “Maintaining Autonomy” was represented in 28.57% (n = 52) of the sample. There was a steep decline in interest for the rest of the recommendations, beginning with “Encouraging Public Involvement”, which was only represented in 14.29 (n = 26) of the sample. “Maintaining Choice” coded for 13.74% (n = 25) of the sample. Finally, “Not For Public Use” and “Not For Sale/Profit” were the least represented recommendations, with only 9.34% (n = 17) and 5.49% (n = 10) of mentions in the sample, respectively.

### 3.5. Tone

**Figure 6:**
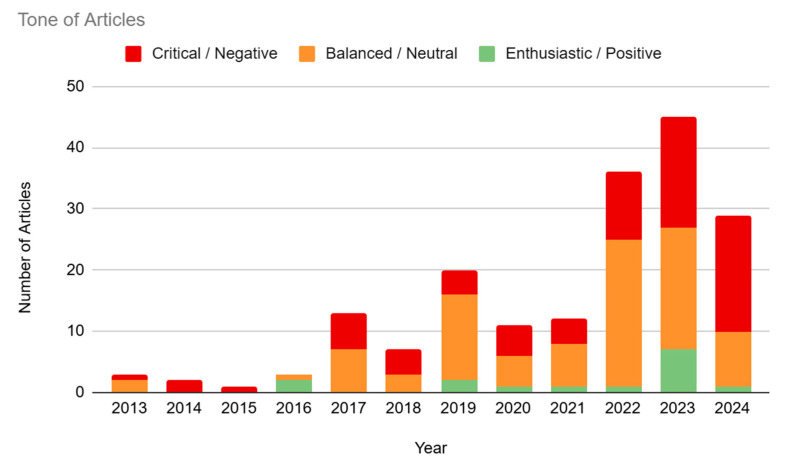
Article Tone.

Despite the general tendency for media articles to fall into “hype and hope” or “gloom and doom” views cf. [27], the majority of the articles present in our sample were coded as “Balanced/Neutral” (50.55%, n = 92). The category of “Negative/Critical” followed closely, with 41.21% (n = 75) of articles expressing more negative views on BCI technology. Finally, only 8.24% (n = 15) of articles were coded as “Positive/Enthusiastic”. There was no clear trend in tone over the years, partially due to a small sample size in 2013–2016, though 2024 did show significantly higher levels of critical articles (65.52%) compared with the rest of the sample (see Figure 6 and Table 2).

### 3.6. Application

**Figure 7:**
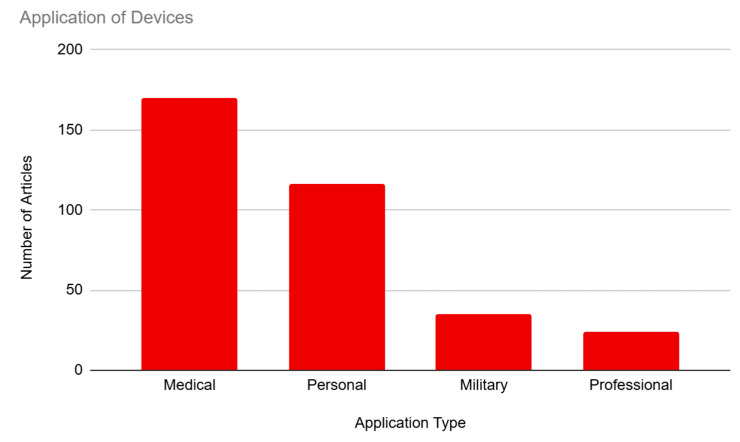
Applications for BCI Devices.

Our articles were coded for the application of BCI technology, which included medical, personal, military, and professional applications (see Figure 7). Medical usage of BCIs was represented most frequently, with 93.41% (n = 170) of articles mentioning the medical benefits of the technology. This was followed by personal usage of the technology, which was mentioned in 63.74% (n = 116) of the sample. There was a significant drop-off in interest in military and professional applications, with military usage featured in 19.23% (n = 35) of the sample and professional usage being mentioned in only 13.19% (n = 24) of the sample.

## 4. Discussion

Our results show that public interest in ethical issues regarding BCI technology is still relatively new. While trends from 2013 to 2016 indicate some public awareness of the technology’s existence, 2017 proved to be the year that ignited a broader discussion about potential ethical issues concerning the devices. There was a clear upward trend in media articles discussing ethics as time went on, barring a slight decrease in reporting in the years 2020 and 2021, likely attributable to the coronavirus pandemic, which disrupted non-urgent research activity. Though our study only covers January through the end of June of 2024, current publication trends indicate that 2024 will likely result in the greatest proliferation of media articles on BCI ethics thus far.

Similar to the academic discussion of BCI ethics [2,9], concerns regarding privacy and safety dominated the ethical concerns. We observed a significant decline in the discussion of our other ethical codes, with most issues being mentioned at less than half the rate of the top two. Medical usage, which was our most prominent application type, was mainly correlated with safety concerns. The majority of these safety concerns revolved around the issue of implantation and discussed the danger inherent in every surgery, emphasizing the risk of undergoing brain surgery for a device that had not yet been perfected. A smaller number of articles also featured discussion of device malfunctions and biohacking endangering the health of patients. Concerns of privacy were primarily discussed in conjunction with personal application of the devices, which reflected public anxieties about companies using the data of consumers to create targeted advertisements or even erode personal autonomy. These anxieties seemed to be tied to the rapidly growing market for BCI companies and research, with several articles citing the presence of Meta and Neuralink in the BCI market as a cause of their concerns.

Neuralink specifically had a profound impact on public discourse on BCI ethics, arguably due to the reputation and name recognition of Elon Musk. Musk has already made a public name for himself due to his previous business ventures including the automotive company Tesla, his space exploration company SpaceX, and his recent acquisition of the social media site “X” [28]. Indeed, the rise in public interest in BCIs is likely due in part to the announcement of Neuralink that took place in 2017 [28]. The recommendation of “Not For Sale/Profit” was only suggested after 2019 and was highly correlated with mentions of Neuralink, and ethical concerns about overpromising were often tied directly to Musk’s public involvement in BCI research. The largest impact of Neuralink on media representation involved an animal testing controversy and subsequent lawsuit by the Physicians’ Committee of Responsible Medicine (PCRM) that occurred in 2021 [29], which resulted in a sudden increase in reporting on the issue of animal welfare. The lawsuit alleged that Neuralink’s animal trials had caused the unnecessary suffering and deaths of several of the macaque monkeys that had been implanted with the devices. A majority of the articles that reported on this issue took on a negative tone in their discussion of BCIs, and most articles mentioning Neuralink after 2021 acknowledged the controversy even if it was not the article’s focus. This negative association followed mentions of Neuralink until the FDA approval of human trials in 2023 [30], after which most articles began to take on a neutral or balanced tone when discussing BCIs and the company.

Despite some of the controversies and subsequent concerns about the safety of BCIs, our study showed that most articles featured a neutral or balanced tone when discussing ethical issues pertaining to the devices. This result is significant considering that media articles have a general tendency to fall into “hype and hope” or “gloom and doom” views, which can have a profound impact on future research [27]. It seems that despite some hesitations, public perception takes on a cautiously optimistic stance. This is reflected in the recommendation of “More Research” being the most frequently coded for, followed by “Regulation”. A majority of articles acknowledged a need for policy and government involvement before the technology reaches a stage in which it is readily available for either medical or public consumption. Notably, many articles cited Chile’s creation of a new constitutional amendment that enshrined the creation of neurorights, thereby protecting its citizens’ private brain data from potential exploitation [31]. Aside from government intervention, many articles also recommended medical oversight in order to protect patients from potential harm.

Generally, articles that discussed patient care approached ethical issues from the lens of care ethics, and acknowledged the importance of trust and consent in doctor–patient relationships. Many of these articles featured stories of individual patients who had suffered debilitating injuries that could potentially be treated with BCI technology and explored the nuances of their relationship with their peers before and after the implantation of the device. The categorical imperative, our second-most coded ethical framework, was also often applied to medical applications of the device. Namely, concerns were raised about exploitation and using patients as a means to an end when conducting research. This ethical lens also lent itself to discussion of personal usage of BCIs, with many articles suspecting that the sudden interest in research by companies, like Meta, were tied to the desire to use individuals’ neural data in order to drive up sales of other products. Articles that focused on the far-reaching implications of the normalization of BCI technology often considered the ethical issues present from a utilitarian lens, speculating on whether the devices would have a net positive or negative impact on society.

Due to human trials of the devices only being in their infancy, military and professional applications of the devices were severely underrepresented. With this being said, articles that featured military usage of BCIs were usually of a critical tone. Concerns involving the intersection of existing political and legal issues with the public availability of the devices (such as accessibility, inequality, and responsibility) were not commonly featured. Unsurprisingly, articles tended to focus on pressing issues that currently affect BCI research or individual consequences of BCI usage rather than how the devices may affect broader institutions. Despite this, there was significant interest in the implications that BCIs may have on personhood and what it means to be human.

Since public opinion has the potential to affect both future regulation and research on BCIs, the media has a responsibility to continue to offer a balanced and unbiased perspective on the devices that serve as accessible ways for the public to stay informed. This is especially pertinent given the conflicts of interest that may arise as social media stakeholders begin to buy into and develop BCI companies of their own. Our data show that public interest in BCI technology is only continuing to grow, and as such, it is important to continue to evaluate the current state of the technology in the public sphere. The majority of the articles (50.55%, n = 92) that we evaluated presented their information to the public in a neutral or balanced manner. Though critical portrayal of BCIs was close behind (41.21%, n = 75), the focus of these articles was predominantly on urgent concerns that BCIs posed, rather than the speculation and fear-mongering that media is often known for. Discussions about privacy reflect the current discourse surrounding the right to data privacy in academic ethics literature [2] and online spaces, and most concerns that were expressed about safety regarding the devices can be attributed to medical research currently being in its infancy.

Nevertheless, it is necessary to foster an environment such that further research and regulation proceed in a way that is beneficial to all parties involved. To start, there should be an increased focus on accessible and accurate information that is disseminated to the public. Academic involvement and collaboration with media publications could help increase public understanding of the more complex issues that arise as the technology continues to develop. Policymakers should also be encouraged to create and maintain a source of reliable information regarding BCIs and encourage public involvement in the discourse. As the academic community recommended with earlier neurotechnologies, increasing (neuro)scientific literacy, fostering an ethical and responsible clinical practice, and monitoring and regulating device use would be the wise course of action see, e.g., [27].

To curtail misunderstandings about BCIs, professional societies, and researchers, perhaps in collaboration with academic publishers, could help the public understand the mechanisms of action BCIs (including active, passive, and reactive forms as well as any additional forms of BCIs that may be in the development phase), contrasting limitations of invasive and wearable devices, and the genuine therapeutic potential of the technology. Governmental agencies, for instance, the Ministry of Health or the Food and Drug Administration, could draft and request public comments on state-of-the-art descriptive information about BCIs to decrease the public’s susceptibility to misinformation. These “fact sheets” could clarify the pertinent risks and benefits, explain the mechanism of action of currently approved BCI devices layman’s terms, and clearly outline what the taxpayer’s money was used for in terms of cutting edge research. This would increase scientific literacy, not only among members of the public, but also clinicians and science journalists writing about BCIs.

Specifically, raising clinicians’ awareness about BCIs would be prudent. Previous clinical responses to emerging neurotechnologies are good examples to emulate. Crucially, clinicians must have and provide patients with accurate information about benefits of BCI devices and potential risks, and potential sources of concern (e.g., the possibility of exclusion from prospective clinical trials, financial costs, etc.). Clarifying the role of clinicians with respect to requests from patients for enhancement applications of BCIs brings additional complexity, since enhancement practices raise distinct ethical, legal and social issues that may lack well-established guidelines in current clinical practice standard operating procedures.

While regulatory agencies such as the FDA govern clinical trials and market approval, there are instances in which ethical violations may have occurred. Policymakers have an obligation to introduce legislation designed to protect their citizens if regulatory oversight is insufficient to discourage such violations. As was seen with the Neuralink lawsuit, ethical violations that occur while a technology is still developing can have a substantial impact on public perception. With this in mind, it is imperative that (neuro)ethicists and policymakers work together in order to establish ethical and legal frameworks to protect those who may be impacted by BCIs.

The regulation of medical devices is a complex area where discrepancies between European, North American, and Asian markets create significant challenges by impacting the dissemination and use of these devices internationally. There is currently no consensus on BCI regulation, though several leading authors have proposed ways to manage new and emerging neurotechnology devices with potential enhancement effects by accommodating them within the current regulations for medical devices [32]. Arguably, a reasonable policy solution should include the monitoring of adverse events stemming from BCIs in addition to paying closer attention to marketing and manufacturing standards. Simple policy actions could be to make mandatory disclaimers about the current evidence supporting BCI use in therapeutic and “wellness” contexts.

## 5. Limitations

Certain aspects of the methodology of this study have the potential to increase the risk of confirmation bias. Since only articles which discussed the ethics of BCI technology made up the sample set, our findings may portray a more negative view of BCI technology in the media. Articles discussing ethics tend to evaluate risks and include more nuanced and critical perspectives, which was recorded in our conclusions on the tone of articles. It may be true that if we did not select only articles that discussed the ethics of BCI technology, more enthusiastic articles would be found.

Previous findings on media depictions of neurotechnology have conversely found a more enthusiastic tone in articles. DBS technology was found to be portrayed very optimistically [33]. Likewise, the media portrayal of BCI technology also appeared to show a more positive tone than our findings identified and that the majority of articles on this topic do not engage with ethics [34]. Our contrasting results reflect the choice to only use articles that discuss ethics. We recognize this potential bias and acknowledge that our findings may not accurately capture the overall tone of all media portrayals of BCI technology.

Additionally, our sample was limited to media articles published in English. While we recognize that valuable work in the realm of BCI is conducted in other world languages, future studies in a diverse range of languages may need to be compared with our results in order to increase generalizability. Additionally, this focus on one language makes comparison between countries fairly difficult, and we refrained from speculating on this front.

## 6. Conclusions

This rapid review analyzed recent media publications in order to clarify where current media perception stands on ethical issues pertaining to BCIs. We determined that while BCIs are most commonly presented with a balanced or neutral tone, critical or negative portrayals of the devices do not lag far behind. A large portion of these ethical concerns and negative portrayals are due to increased news coverage of emergent companies such as Neuralink. The most common issues discussed were that of privacy and safety, likely reflecting a distrust in corporate misuse of private information and concerns about medical research of the devices still being in its infancy. A focus on care ethics as an ethical framework indicates that there is an interest in how BCIs may affect the interpersonal relationships of its users. There is a general consensus that more research and regulation are needed in order to minimize harm as BCIs become more prominent. It is important that ethical discussions regarding BCIs remain open and accessible to the public in order to prevent the spread of misinformation.

## Figures and Tables

**Table 1:** Illustrations of Ethical Issues Quoted in Media Sample.

Ethical Issues	Quotation from Media Sample Texts
Biohacking	“One scary scenario could involve users being controlled and unconsciously acting because they cannot decide if their actions are ethical or even true. They could steal the possessions of others or, in the worst-case, kill.”
Autonomy	“When referring to authenticity, the team means to the extent to which an individual feels that their abilities and accomplishments are their own, even if those abilities are augmented by the brain implant, or their accomplishments were made with the assistance of BCI technologies.”
Coercion/Consent	Will this decision be truly and solely ours to make?What I’m worried about the most—and what you need to consider as well—is whether we’ll even have an actual choice to ‘opt out’ if BCIs are implemented.
Privacy	“Any system that could collect data directly from our brains has clear privacy risks.”
Weaponization	“Enhancing soldiers might create the greatest good by improving a nation’s warfighting abilities, protecting military assets by keeping soldiers remote, and maintaining military readiness.”
Safety	We would also recommend that the U.S. Government work through the existing regulatory bodies, like the FDA, to oversee the safety and security of these devices, especially those invasive BCI devices that are used for health care applications.
Animal Welfare	“However, the company’s previous animal testing has come under scrutiny following reports of unnecessary suffering. Former employees have described these tests as substandard, with one instance involving the improper placement of the device in pigs, leading to their euthanasia.”
Responsibility	Adina Roskies, professor of philosophy at Dartmouth University, says that while such a “cyborg future” might seem compelling, it raises thorny ethical questions around identity and moral responsibility.
Personhood/Identity	“Well, I for one wouldn’t be standing in line waiting for my brain implant, as it would take away too much of what makes me who I am.”
Accessibility	“Reported costs of wearable BCIs range from hundreds to thousands of dollars, which may result in unequal access.”
Inequality	“In this sense, it is necessary to regulate technology to avoid inequality between people.”
Overpromising	“As a scientist myself, I look forward to the day when the technology Musk describes can solve these problems. Yet every one with basic natural science training knows how many difficulties one has to overcome to turn “in principle” into practice.”
Exploitation	“She is referring not only to potential mental and physiological side effects that have yet to be explored, but also to the potential for exploitation of users’ neural data by the private companies developing these technologies.”
Regulation	“Despite the necessary regulations, in the virtual presentation of the legislative plan, organized yesterday from the Chilean capital, there was consensus on the goodness of neurotechnology.”

**Table 2:** Illustrations of Tone Indicators in Media Sample.

Tone Indicated	Quotation from Media Sample Texts
**Positive/Enthusiastic**	“BCI involves technology even more advanced than artificial intelligence. It has just started to grow,” Yu said. “Although it is so far largely unknown to the public, there is huge potential.”
**Neutral/Balanced**	“We can’t anticipate or solve all of the ethical issues associated with this technology on our own. What we can do is recognise when the technology has advanced beyond what people know is possible, and make sure that information is delivered back to the community,” said Mark Chevillet, director of the brain-computer interface program at Facebook Reality Labs.“Neuroethical design is one or our programme’s key pillars -we want to be transparent about what we’re working on so that people can tell us their concerns about this technology.”
**Negative/Critical**	“Musk admits that he takes inspiration from science fiction, and that he wants to make sure humans can “keep up” with artificial intelligence. He seems to have missed the part of sci-fi that acts as a warning for the implications of technology.These mind-reading systems could affect our privacy, security, identity, equality and personal safety. Do we really want all that left to companies with philosophies such as that of Facebook’s former mantra, “move fast and break things”?”

## Data Availability

Data supporting reported results, including links to datasets analyzed during the study, are available from the corresponding author. The data is public but behind a paywall. Readers who do not have access can contact the corresponding author.

## Supplementary Materials

The following supporting information can be downloaded at https://www.mdpi.com/article/10.3390/brainsci14121255/s1: Table S1: The Full List of Media Articles.
