# Supplementary material for: Media Representation of the Ethical Issues Pertaining to Brain–Computer Interface (BCI) Technology
Source: Brain Sci. 2024 Dec 14;14(12):1255. doi: 10.3390/brainsci14121255 (PMC11674794; doi:10.3390/brainsci14121255)
Supplement: Supplementary file 1 [file brainsci-14-01255-s001.zip › brainsci-3315989-supplementary.pdf]

Table S1: The Full List of Media Articles

Publisher	Authors	Title	Year	Ethical Issues	Ethical Frameworks	Recommendations	Tone	Application
ENP Newswire	Editorial Staff	-KINGS COLLEGE LONDON -New brain treatments: proceed with care	2013 (1)	1) Coercion / Consent; 2) Safety; 3) Overpromising	Care Ethics	1) Avoiding undesired results; 2) Regulation; 3) More research; 4) Medical oversight	Balanced/Neutral	Medical; Personal
MailOnline	Ellie Zolfagharifard	Darth Vader eat your heart out: Researcher controls a colleague's hand by channelling his brain signals through the INTERNET	2013 (4)	1) Autonomy; 2) Weaponization	Care Ethics	1) More research	Balanced/Neutral	Medical
Activist Post	Nicholas West	7 Future Methods of Mind Control	2013 (8)	1) Biohacking; 2) Weaponization; 3) Personhood / Identity	Categorical Imperative	1) Avoiding undesired results; 2) Regulation; 3) Maintaining choice; 4) Maintaining autonomy	Negative/Critical	Military
Activist Post	Nicholas West	The Era Of Cyborgs Has Begun	2014 (1)	1) Privacy; 2) Personhood / Identity	Other	1) Regulation; 2) Maintaining autonomy; 3) Not for public use; 4) Avoiding undesired results	Negative/Critical	Medical; Personal
Activist Post	Nicholas West	Mind Control Scientists Using Light To Alter The Brain	2014 (4)	1) Biohacking; 2) Autonomy	Categorical Imperative	1) Regulation; Maintaining autonomy	Negative/Critical	Medical

Activist Post	Nicholas West	Nanoparticles Enable Remote Control Brains Via Magnetic Field: New Study	2015 (6)	1) Biohacking; 2) Autonomy; 3) Weaponization	Categorical Imperative	1) Regulation; 2) Maintaining choice; 3) Maintaining Autonomy; 4) Not for public use	Negative/Critic al	Medical; Military

The Christian Science Monitor	Ben Thompson	Will 'mind-controlled' drones be the newest way to fly?; A recent thought-powered drone race could signal a first step in brain-controlled technology becoming more usable and widespread in everyday life.	2016 (4)	1) Biohacking; 2) Weaponization	1) Utilitarianism	1) Encouraging public involvement	Positive/Enthusia stic	Medical; Personal; Military
The Independent	Jason Dearen	It's blue-sky thinking: brain-powered drones are taking to the air '	2016 (15)	1) Privacy; 2) Weaponization	1) Other	1) Encouraging public involvement; 2) Avoiding undesired results	Positive/Enthusia stic	Medical; Personal
European Union News	Editorial Staff	Register of Commission documents: Contract rules for online purchase of digital content and tangible goods (Part of Digital Single Market): Implementation Appraisal Document date: 2016-04-08 EPRS_BRI(2016)573293 Briefing	2016 (22)	1) Coercion/Consent; 2) Privacy; 3) Safety; 4) Responsibility; 5) Inequality; 6) Regulation	1) Care ethics; 2) Categorical Imperative	1) Avoiding undesired results; 2) Regulation; 3) More research	Balanced/Neutral	1) Medical; 2) Personal
	Premium Official News	Ethical issues surrounding implantable brain technologies leads academic to US	2017 (1)	1) Autonomy 2) Personhood/Identi ty	1) Care Ethics	1) Encouraging public involvement; 2) More research; 3) Maintaining autonomy	Negative/Critical	1) Medical

GeekWire	Chelsey Ballarte	EXPERTS LAY OUT THEIR CONCERNS ABOUT THE ETHICS OF BRAIN IMPLANTS AND BRAINJACKING'	2017 (2)	1) Biohacking; 2) Autonomy; 3) Responsibility	1) Utilitarianism; 2) Care Ethics	1) Avoiding undesired results; 2) Regulation; 3) Maintaining oversight	Negative/Critical	1) Medical; 2) Personal
Medical Xpress	Wys Center For Bio and Neuroenginering	Who is responsible if a brain-controlled robot drops a baby?	2017 (3)	1) Biohacking; 2) Responsibility; 3) Privacy; 4) Overpromising	1) Care Ethics	1) Avoiding undesired results; 2) Regulation	Negative/Critical	1) Medical; 2) Personal
MailOnline	Eran Klein; Katherine Pratt	What could possibly go wrong... Researchers call for urgent research into the risks of DIY brain hacking	2017 (4)	1) Biohacking; 2) Coercion/Consent; 3) Privacy	1) Categorical Imperative	1) Encouraging public involvement; 2) Avoiding undesired results; 3) Regulation	Negative/Critical	1) Medical; 2) Personal
SharpBrains	Laurey Bartels	Responsible neuroengineering: Neurotechnology and artificial intelligence (AI) can enhance our lives if it preserves our 1) privacy, 2) identity, 3) agency and 4) equality, researchers say	2017 (6)	1) Privacy; 2) Responsibility	1) Care Ethics	1) Regulation	Negative/Critical	1) Medical; 2) Personal
MailOnline	Harry Pettit	Computers of the future could monitor, store and DELETE your thoughts without you ever knowing	2017 (11)	1) Biohacking; 2) Privacy; 3) Regulation	1) Care Ethics; 2) Categorical Imperative	1) Avoiding undesired results; 2) Regulation	Negative/Critical	1) Personal
I4U News	Sumayah Aamir	Elon Musk Launches Neuralink to Read Human Brains With Computers	2017 (12)	1) Safety	1) Care Ethics	1) More research; 2) Medical oversight	Balanced/Neutral	1) Medical; 2) Personal

BGR: Your Mobile Life	Deepali Moray	Elon Musk's new venture wants to hack into the human brain using AI	2017 (13)	1) Safety	1) Care Ethics	1) More research; 2) Medical oversight	Balanced/Neutral	1) Medical; 2) Personal
Atlantic Online	Rachel Mabe	What Is It Like to Regain a Sense of Touch, Only to Lose It Again?	2017 (16)	1) Safety	1) Care Ethics; 2) Categorical Imperative	1) Medical oversight; 2) Maintaining choice	Balanced/Neutral	1) Medical
i-Independent Print Ltd	Rhiannon Williams	Facebook's new brainwave; Technology that turns our thoughts into words will be life-changing - and it's closer than we realise	2017 (19)	1) Privacy; 2) Personhood/Identity	1) Utilitarianism	1) Avoiding undesired results; 2) Regulation; 3) Maintaining autonomy	Balanced/Neutral	1) Personal
Voice of America	Elizabeth Lee	Brain-computer Connection Unlocks Possibilities, Both Good and Bad	2017 (21)	1) Biohacking; 2) Privacy; 3) Regulation	1) Utilitarianism	1) Regulation	Balanced/Neutral	1) Medical; 2) Personal
CNN	Jacqueline Howard	Decoding the thoughts of patients who can't even blink	2017 (29)	1) Autonomy; 2) Coercion/Consent	1) Care Ethics	1) More research; 2) Medical oversight	Balanced/Neutral	1) Medical
Sunday Age	Paul Biegler	Elon Musk wants to upgrade your brain	2017 (37)	1) Autonomy; 2) Personhood/Identity	1) Utilitarianism	1) Avoiding undesired results; 2) Regulation; 3) Maintaining autonomy	Balanced/Neutral	1) Medical; 2) Personal; 3) Military

UW News	Sarah McQuate	UW-LED PHILOSOPHY TEAM RECEIVES $1.5M GRANT TO STUDY THE ETHICS OF NEUROTECHNOLOGY RESEARCH	2018 (6)	1) Coercion/Consent; 2) Responsibility	1) Care Ethics; 2) Categorical Imperative	1) More research; 2) Maintaining choice	Balanced/Neutral	1) Medical
The Daily Gleaner	Editorial Staff	What's the emoji for curmudgeon?	2018 (16)	1) Privacy; 2) Weaponization	1) Other	1) Avoiding undesired results; 2) Maintaining autonomy	Negative/Critical	1) Medical; 2) Personal; 3) Military; 4) Professional
TheNextWeb.com	Gerd Leonhard	Brain implants are happening — are you ready for yours?	2018 (17)	1) Autonomy; 2) Coercion/Consent; 3) Weaponization; 4) Personhood/Identity; 5) Inequality; 6) Regulation	1) Other	1) Avoiding undesired results; 2) Maintaining autonomy	Negative/Critical	1) Medical; 2) Personal; 3) Military; 4) Professional
Wired	Editorial Staff	PLUGGED IN	2018 (23)	1) Coercion/Consent; 2) Inequality; 3) Accessibility	1) Categorical Imperative	1) Avoiding undesired results	Negative/Critical	None
Sydney Morning Herald	Simon McCarthy-Jones, Susie Alegre	Social media threatens right to think	2018 (30)	1) Biohacking; 2) Autonomy; 3) Privacy	1) Categorical Imperative	1) Avoiding undesired results; 2) Regulation; 3) Maintaining autonomy	Negative/Critical	1) Personal
The Atlantic	Michael Joseph Gross	The Pentagon's Push to Program Soldiers' Brains	2018 (34)	1) Autonomy; 2) Coercion/Consent; 3) Weaponization; 4)	1) Categorical Imperative	1) Avoiding undesired results; 2) Regulation; 3) More research; 4) Maintaining autonomy	Balanced/Neutral	1) Medical; 2) Military

				Personhood/Identity; 5) Inequality				
Conservative Daily News	Tim Vernimmen	Bypassing paralysis	2018 (37)	1) Safety; 2) Accessibility; 3) Inequality	1) Care Ethics	1) Avoiding undesired results; 2) Regulation; 3) More research; 4) Not for public use	Balanced/Neutral	1) Medical; 2) Personal
News Chronicle, Nigeria.	Akanimo Sampson	A Neural Implant Can Access Your Brain Through the Jugular Vein	2019 (1)	1) Privacy; 2) Responsibility	1) Care Ethics	1) More research; 2) Medical oversight	Balanced/Neutral	1) Medical; 2) Personal
The Conversation, UK	Garfield Benjamin	Silicon Valley wants to read your mind - here's why you should be worried	2019 (3)	1) Autonomy; 2) Privacy; 3) Safety; 4) Accessibility; 5) Inequality	1) Utilitarianism; 2) Care Ethics	1) Avoiding undesired results; 2) Regulation; 3) More research; 4) Maintaining choice; 5) Maintaining autonomy	Negative/Critical	1) Medical; 2) Personal; 3) Professional
Global Times, China	Leng Shumei	SpaceX CEO brain-chip plan wows Chinese internet users	2019 (5)	1) Safety	1) Care Ethics	1) Avoiding undesired results; 2) More research; 3) Medical oversight	Balanced/Neutral	1) Medical; 2) Personal
Financial Times, UK	Alice Hancock	Forget brain chips, live experiences are the future of entertainment	2019 (11)	1) Safety	1) Other	1) Not for public use	Balanced/Neutral	1) Medical; 2) Personal

NPR Morning Edition	Elise Hu; David Greene	VIDEO: Elon Musk's Next Quest Is A Mind-Machine Meld. Let's Consider The Implications	2019 (14)	1) Personhood/Identity	1) Other	1) Avoiding undesired results; 2) More research	Balanced/Neutral	1) Medical; 2) Personal
Medical Xpress	Roger Gassert	Tricky interfaces: Brain-computer interfaces are still a long way off	2019 (16)	1)Biohacking; 2) Privacy; 3) Safety; 4) Responsibility; 5) Inequality	1) Care Ethics; 2) Categorical Imperative	1) Avoiding undesired results; 2) Regulation; 3) More research	Balanced/Neutral	1) Medical; 2) Personal
China Daily European Edition	Zhou Mo, Xin Wen	Future of high-tech may rely more on brain power	2019 (18)	1) Safety	1) Care Ethics	1) More research; 2) Medical oversight	Positive/Enthusiastic	1) Medical; 2) Personal; 3) Military; 4) Professional
ReadWrite	Frank Landman	Are Cyborgs Already Here? An Intro to the Debate and Why It Matters	2019 (19)	1) Autonomy; 2) Privacy; Personhood/Identity; 3) Inequality; 4) Regulation	1) Categorical Imperative	1) Encouraging public involvement; 2) Regulation; 3) More research; 4) Maintaining autonomy	Balanced/Neutral	1) Personal; 2) Professional
New York Observer	Harmon Leon	Telepathy Tech Is the Latest Sci-Fi Marvel to Emerge in the Real World	2019 (23)	1) Autonomy; 2) Privacy; 3) Weaponization; 4) Personhood/Identity	1) Categorical Imperative	1) Avoiding undesired results; 2) Maintaining autonomy	Negative/Critical	1) Medical; 2) Personal; 3) Military
Business Monitor Online	Editorial Staff	Brain-Computer Interface Technology Could Provide Medical Breakthroughs	2019 (24)	1) Biohacking; 2) Safety	1) Care Ethics; 2) Categorical Imperative	1) Avoiding undesired results; 2) Regulation; 3) More research	Balanced/Neutral	1) Medical; 2) Personal

The Observer, London	Zoë Corbyn	Are brain implants the future of thinking?; Brain-computer interface technology is moving fast and Silicon Valley is moving in. Will we all soon be typing with our minds?	2019 (31)	1) Biohacking; 2) Privacy; 3) Safety; 4) Responsibility; 5) Accessibility	1) Categorical Imperative	1) Avoiding undesired results; 2) Regulation; 3) Maintaining choice	Balanced/Neutral	1) Medical; 2) Personal; 3) Military
The Independent, UK	Anthony Cuthbertson	Brain-computer interface will make people telepathic, scientists say; People will communicate 'not only without speaking but without words -through access to each other's thoughts at a conceptual level'	2019 (33)	1) Privacy; 2) Personhood/Identity	1) Other	1) More research	Positive/Enthusiastic	1) Medical; 2) Personal
The Telegraph, UK	Harry de Quetteville	Is the robotic suit the first step on the slippery slope to us all becoming cyborgs?	2019 (35)	1) Personhood/Identity	1) Other	1) Avoiding undesired results; 2) Maintaining autonomy	Negative/Critical	1) Medical; 2) Personal; 3) Military; 4) Professional
The Independent, UK	Anthony Cuthbertson	Facebook says it can read thoughts with mind-reading device; 'We're standing on the edge of the next great wave in human-orientated computing,' Facebook says	2019 (41)	1) Privacy	1) Categorical Imperative	1) Encouraging public involvement	Balanced/Neutral	1) Medical; 2) Personal
CNN Wire, San Francisco	Rachel Metz	Elon Musk hopes to put a computer chip in your brain. Who wants one?	2019 (44)	1) Biohacking; Safety	1) Care Ethics	1) Avoiding undesired results; 2) Regulation; 3) More research; 4) Medical oversight	Balanced/Neutral	1) Medical; 2) Personal
The Guardian, London	Oscar Schwartz	Mind-reading tech? How private companies could gain access to our brains; Social media companies can already use online data to make reliable guesses about pregnancy or suicidal ideation - and new BCI technology will push this even further	2019 (45)	1) Privacy, 2) Responsibility; 3) Personhood/Identity; 4) Regulation	1) Care Ethics; 2) Categorical Imperative	1) Avoiding undesired results; 2) Regulation; 3) More research	Balanced/Neutral	1) Medical; 2) Personal

South China Morning Post, China	Celia Chen	All you need to know about the brain-computer interface, the technique Elon Musk wants to use to merge man and machines	2019 (47)	1) Biohacking; 2) Safety	1) Care Ethics	1) Avoiding undesired results; 2) More research	Balanced/Neutral	1) Medical; 2) Personal
Brisbane Times	Paul Biegler	Could big tech companies be holding the keys to our thoughts?	2019 (54)	1) Coercion/Consent; 2) Privacy; 3) Personhood/Identity; 4) Regulation	1) Care Ethics; 2) Categorical Imperative	1) Avoiding undesired results; 2) Regulation; 3) Maintaining choice; 4) Not for public use; 5) Not for sale/profit	Balanced/Neutral	1) Medical; 2) Personal; 3) Professional
Activist Post	Editorial Staff	DARPA Wants to Create Mind-Controlled Weapons of War	2019 (55)	1) Weaponization; 2) Personhood/Identity	1) Care Ethics; 2) Categorical Imperative	1) Maintaining choice; 2) Maintaining autonomy	Negative/Critical	1) Military
The Verge	Casey Newton	Brain-computer interfaces are developing faster than the policy debate around them	2019 (61)	1) Privacy	1) Care Ethics; 2) Categorical Imperative	1) Encouraging public involvement; 2) Avoiding undesired results; 3) Regulation; 4) More research	Balanced/Neutral	None
NCSU Press Re[illegible]	Editorial Staff	Studies Outline Key Ethical Questions Surrounding Brain-Computer Interface Tech	2020 (1)	1) Safety; 2) Animal Welfare; 3) Personhood/Identity	1) Care Ethics	1) Encouraging public involvement; 2) Avoiding undesired results; 3) Regulation	Balanced/Neutral	1) Medical; 2) Personal; 3) Professional
Gozde Bayar	Anadolu Agency	IT expert issues warning on computer brain chips	2020 (7)	1) Biohacking; 2) Autonomy; 3) Safety	1) Categorical Imperative	1) Avoiding undesired results; 2) Medical oversight; 3)Maintaining autonomy	Negative/Critical	1) Medical; 2) Personal
MailOnline	Stacy Liberator	Elon Musk's Neuralink and other brain chips spark ethical concerns among experts who say implants could be seen as 'smart drugs' and testing on animals 'presents moral dilemmas'	2020 (8)	1) Animal welfare; 2) Responsibility; 3) Personhood/Identity; 4) Inequality	1) Care Ethics; 2) Categorical Imperative	1) Avoiding undesired results; 2) Regulation; 3) More research; 4) Maintaining choice	Negative/Critical	1) Medical; 2) Personal

Relias Media	Editorial Staff	Neurotechnology Takes Human Research Ethics to New Frontiers	2020 (9)	1) Autonomy; 2) Safety; 3) Responsibility; 4) Accessibility	1) Care Ethics; 2) Categorical Imperative	1) Encouraging public involvement; 2) Avoiding undesired results; 3) More research; 4) Medical oversight	Negative/Critical	1) Medical; 2) Military
China Daily European Edition	Yuan Lanfeng	Still lot of questions to be answered about brain-computer interface devices	2020 (12)	1) Safety; 2) Overpromising	1) Care Ethics	1) More research; 2) Medical oversight	Balanced/Neutral	1) Medical
RAND Corporation	Marissa Norris	BRAIN-COMPUTER INTERFACES ARE COMING. WILL WE BE READY?	2020 (13)	1) Biohacking; 2) Autonomy; 3) Privacy; 4) Weaponization; 5) Safety	1) Categorical Imperative	1) Regulation; 2) More research; 3) Maintaining autonomy; 4) Not for public use	Balanced/Neutral	1) Medical; 2) Personal; 3) Professional; 4) Military
Khaleej Times, UAE	Aradhana Sharma	Machines in human brains is a terrifying thought	2020 (14)	1) Autonomy; 2) Privacy; 3) Inequality	1) Categorical Imperative	1) Encouraging public involvement; 2) Avoiding undesired results; 3) Regulation; 4) Maintaining autonomy	Negative/Critical	1) Medical; 2) Personal
CE Noticias Financieras English	Editorial Staff	Chile, global neuro-right laboratory	2020 (29)	1) Autonomy; 2) Privacy; 3) Personhood/Identity; 4) Accessibility; 5) Inequality; 6) Regulation	1) Utilitarianism; 2) Care Ethics	1) Avoiding undesired results; 2) Regulation; 3) Maintaining autonomy	Negative/Critical	1) Medical; 2) Personal

The New York Times	Moises Velasquez-Manoff	The Brain Implants That Could Change Humanity	2020 (37)	1) Biohacking; 2) Autonomy; 3) Coercion/Consent; 4) Privacy; 5) Personhood/Identity; 6) Inequality	1) Care Ethics; 2) Categorical Imperative	1) Avoiding undesired results; 2) Regulation; 3) More research	Balanced/Neutral	1) Medical; 2) Personal
Newstex Blogs	Fiona J McEvoy	How Silicon Valley wants to fuck with our brains	2020 (41)	1) Autonomy; 2) Privacy; 3) Weaponization	1) Categorical Imperative	1) Encouraging public involvement; 2) Avoiding undesired results; 3) Regulation; 4) More research; 5) Maintaining autonomy	Balanced/Neutral	1) Medical; 2) Personal; 3) Professional; 4) Military
The Australian Magazine	Cameron Stewart	MIND CONTROL	2020 (43)	1) Biohacking; 2) Coercion/Consent	1) Care Ethics	1) More research; 2) Medical oversight	Enthusiastic	1) Medical; 2) Personal; 3) Professional
Medical Xpress	Editorial Staff	Bleak cyborg future from brain-computer interfaces if we're not careful	2021 (3)	1) Privacy; 2) Personhood/Identity; 3) Inequality	1) Care Ethics	1) Encouraging public involvement; 2) Avoiding undesired results; 3) Regulation	Negative/Critical	1) Medical; 2) Personal
Electronics For You	Deepak Halan	The Future: Brain-To-Brain Communication: Now a Reality	2021 (6)	1) Coercion/Consent; 2) Privacy; 3) Weaponization; 4) Safety; 5) Responsibility; 6) Personhood/Identity; 7) Overpromising	1) Utilitarianism; 2) Care Ethics	1) Regulation; 2) More research; 3) Maintaining choice; 4) Maintaining autonomy	Balanced/Neutral	1) Medical; 2) Personal; 3) Military

Financial Times, London	John Thornhill	Neurotech must not treat humans like mice	2021 (10)	1) Autonomy; 2) Accessibility; 3) Inequality; 4) Regulation	1) Categorical Imperative	1) Avoiding undesired results; 2) Regulation; 3) More research; 4) Maintaining autonomy	Negative/Critical	1) Medical; 2) Personal; 3) Military
The Journal, Newcastle, UK	Ron Beadle	Are you ready to be a cyborg? Let's talk about it first...	2021 (15)	1) Coercion/Consent; 2) Inequality; 3) Regulation	1) Categorical Imperative	1) Encouraging public involvement; 2) Avoiding undesired results; 3) Regulation	Balanced/Neutral	1) Medical; 2) Professional
The Bangkok Post, Thailand	James Hein	The benefits and risks of neural interfaces	2021 (19)	1) Autonomy; 2) Coercion/Consent	1) Categorical Imperative	1) Avoiding undesired results; 2) Maintaining autonomy	Balanced/Neutral	1) Medical; 2) Personal; 3) Military
MailOnline	Ryan Morrison	Brain chips to improve mental health and exoskeletons to make us stronger: Major study predicts humans will use technology to 'upgrade' our lives and ourselves over the next 10 years	2021 (31)	1) Personhood/Identity; 2) Accessibility; 3) Inequality; 4) Regulation	1) Utilitarianism; 2) Care Ethics	1) Avoiding undesired results; 2) Regulation; 3) More research; 4) Maintaining choice; 5) Not for sale/profit	Balanced/Neutral	1) Medical; 2) Personal; 3) Military
Sunday Independent	Patrice Harrington	THEBRAIN CHANGER; Limerick-born neuroscientist Dr Phil Kennedy's mission, to help his patients speak, led him to experiment on himself. In a new film, he explains his belief that creating cyborgs is the future of humanity	2021 (32)	1) Weaponization; 2) Safety; 3) Accessibility	1) Utilitarianism; 2) Care Ethics	1) Avoiding undesired results; 2) More research; 3) Medical oversight; 4) Maintaining choice; 5) Not for sale/profit	Balanced/Neutral	1) Medical; 2) Military

Newstex	Gary Grossman	Thought-detection: AI has infiltrated our last bastion of privacy	2021 (34)	1) Biohacking; 2) Privacy	1) Categorical Imperative	1) Avoiding undesired results; 2) Maintaining choice; 3) Maintaining autonomy	Balanced/Neutral	1) Medical; 2) Personal; 3) Professional
Irish Daily Mail	Philip Nolan	The DOCTOR who HACKED his own BRAIN; Irish neurosurgeon Phil Kennedy risked brain damage and even death in a bid to further his incredible research	2021 (37)	1) Weaponization; 2) Safety	1) Care Ethics	1) More research; 2) Medical oversight	Positive/Enthusiastic	1) Medical; 2) Military
The New Yorker	Christine Kenneally	Mind Machines	2021 (45)	1) Autonomy; 2) Coercion/Consent; 3) Personhood/Identity; 4) Regulation	1) Utilitarianism; 2) Care Ethics	1) Regulation; 2) More research; 3) Medical oversight; 4) Maintaining choice; 5) Maintaining autonomy; 6) Not for sale/profit	Balanced/Neutral	1) Medical; 2) Personal
The New Zealand Herald	Damien Venuto	Brain chips and the death of visas: What work could look like in 2030	2021 (47)	1) Inequality; 2) Regulation	1) Categorical Imperative	1) Avoiding undesired results; 2) Regulation; 3) Maintaining autonomy; 4) Not for sale/profit	Negative/Critical	1) Medical; 2) Personal; 3) Professional
The Geller Report	Pamela Geller	The 'Spartacus COVID Letter' That's Gone Viral: 'Damn You To Hell, You Will Not Destroy America'	2021 (51)	1) Biohacking; 2) Autonomy; 3) Privacy; 4) Weaponization; 5) Personhood/Identity; 6) Inequality	1) Categorical Imperative	1) Avoiding undesired results; 2) Maintaining choice; 3) Maintaining autonomy; 4) Not for public use	Negative/Critical	1) Military
Neuroscience Bulletin	Christopher R. Wood; Yongmei X;, Wei-Jun Yang; Hao Wang	Insight into Neuroethical Considerations of the Newly Emerging Technologies and Techniques of the Global Brain Initiatives	2022 (1)	1) Autonomy; 2) Privacy; 3) Weaponization; 4) Safety; 5) Animal Welfare; 6) Responsibility; 7)	1) Utilitarianism; 2) Care Ethics; 3) Categorical Imperative	1) Avoiding undesired results; 2) Regulation; 3) More research; 4) Maintaining autonomy; 5) Not for public use	Balanced/Neutral	1) Medical; 2) Personal; 3) Military

				Personhood/Identity; 8) Inequality; 9) Regulation				
Fast Company	Nancy S. Jecker; Andrew Ko	Brain-computer interfaces could change the world—but at what cost?	2022 (2)	1) Biohacking; 2) Autonomy; 3) Privacy; 4) Weaponization; 5) Personhood/Identity; 6) Regulation	1) Utilitarianism	1) Avoiding undesired results; 2) Regulation; 3) Maintaining autonomy	Balanced/Neutral	1) Medical; 2) Personal; 3) Professional; 4) Military
The Independent, UK	Anthony Cuthbertson	Elon Musk's Neuralink admits to killing eight animals during brain chip tests	2022 (4)	1) Animal Welfare	1) Care Ethics	1) More research; 2) Medical oversight	Negative/Critical	1) Medical; 2) Personal
The Times, London	Editorial Staff	Elon Musk's attempt to save humanity from the rise of the machines	2022 (7)	1) Safety; 2) Animal Welfare; 3) Overpromising	1) Care Ethics	1) More research; 2) Medical oversight	Negative/Critical	1) Medical; 2) Personal
CE Noticias Financieras English	Editorial Staff	Elon Musk's Neuralink Project is of great concern to scientists	2022 (8)	1) Overpromising; 2) Exploitation	1) Care Ethics; 2) Categorical Imperative	1) Avoiding undesired results; 2) Regulation; 3) Not for public use; 4) Not for sale/profit	Negative/Critical	1) Medical; 2) Personal
US Fed News	Editorial Staff	SCIENCE & TECH SPOTLIGHT: BRAIN-COMPUTER INTERFACES	2022 (9)	1) Biohacking; 2) Coercion/Consent; 3) Weaponization; 4) Safety; 5) Responsibility; 6) Accessibility; 7) Inequality	1) Care Ethics	1) Avoiding undesired results; 2) Regulation; 3) More research; 4) Maintaining autonomy	Balanced/Neutral	1) Medical; 2) Personal; 3) Professional; 4) Military

InnovationAus	Brandon How	Melbourne Uni spin out beats Elon Musk to US clinical trials	2022 (11)	1) Autonomy; 2) Privacy	1) Care Ethics	1) Avoiding undesired results; 2) Medical oversight	Positive/Enthusiastic	1) Medical; 2) Personal
CE Noticias Financieras English	Editorial Staff	Evelyn Melo: For the protection of neuro-rights	2022 (12)	1) Privacy; 2) Personhood/Identity; 3) Accessibility; 4) Inequality; 5) Regulation	1) Care Ethics; 2) Categorical Imperative	1) Avoiding undesired results; 2) Regulation; 3) More research; 4) Maintaining autonomy; 4) Not for public use	Balanced/Neutral	1) Medical; 2) Personal
thestar.com	Kevin Jiang	Neuralink, Elon Musk's secretive brain chip company, streams 'show and tell' update	2022 (14)	1) Animal Welfare	1) Care Ethics	1) More research; 2) Medical oversight	Balanced/Neutral	1) Medical; 2) Personal
CE Noticias Financieras English	Editorial Staff	Elon Musk's Neuralink defends itself against animal abuse claims after criticism	2022 (15)	1) Safety; 2) Animal Welfare	1) Care Ethics	1) More research; 2) Medical oversight	Balanced/Neutral	1) Medical
New York Observer	Sissi Cao	Elon Musk's Brain Chip Company Neuralink Reveals Progress and Is Met With Skepticism	2022 (18)	1) Safety; 2) Overpromising	1) Care Ethics	1) Avoiding undesired results; 2) More research; 3) Medical oversight	Balanced/Neutral	1) Medical

Impact News Service	Kelly Servick	Brain surgeries are opening windows for neuroscientists, but ethical questions abound	2022 (19)	1) Coercion/Consent; 2) Safety; 3) Exploitation	1) Care Ethics; 2) Categorical Imperative	1) More research; 2) Medical oversight; 3) Maintaining choice	Balanced/Neutral	1) Medical
The Independent, UK	Vishwam Sankaran	Elon Musk 'confident' Neuralink brain chip is 'ready for humans'	2022 (20)	1) Animal Welfare	1) Care Ethics	1) More research; 2) Medical oversight	Balanced/Neutral	1) Medical; 2) Personal
mirror.co.uk	Charlie Jones	Elon Musk expects to have Neuralink brain chip in humans in just six months	2022 (21)	1) Animal Welfare	1) Care Ethics	1) More research; 2) Medical oversight	Negative/Critical	1) Medical
CE Noticias Financieras English	Editorial Staff	Two experiments achieve remote control of devices with the mind and without implants	2022 (22)	1) Coercion/Consent; 2) Privacy; 3) Safety; 4) Inequality; 5) Regulation	1) Care Ethics	1) Encouraging public involvement; 2) Regulation; 3) Maintaining choice	Balanced/Neutral	1) Medical; 2) Personal
MailOnline	Ryan Morrison	Elon Musk-owned Neuralink confirms monkeys died during tests for a project to implant computer chips into their brains, but denies claims of animal abuse	2022 (23)	1) Animal Welfare	1) Care Ethics; 2) Other	1) Avoiding undesired results; 2) Regulation; 3) More research	Negative/Critical	1) Medical; 2) Personal
The Verge	Adi Robertson	Elon Musk's brain implant startup Neuralink denies that researchers abused monkeys	2022 (24)	1) Animal Welfare	1) Care Ethics	1) Regulation; 2) More research; 3) Medical oversight	Negative/Critical	1) Medical; 2) Personal

USA Today	Bailey Schulz	Elon Musk's Neuralink wants people to control computers with their minds. How close are they?	2022 (25)	1) Animal Welfare	1) Care Ethics	1) More research; 2) Medical oversight; 3) Not for public use	Balanced/Neutral	1) Medical; 2) Personal
Business Day, South Africa	Johan Steyn	STATE OF THE SMART - AI health care will turn us into different beings	2022 (26)	1) Accessibility; 2) Inequality	1) Care Ethics	1) Regulation; 2) More research; 3) Not for sale/profit	Negative/Critical	1) Medical
CE Noticias Financieras English	Editorial Staff	Brain implant allows totally paralyzed patient to communicate	2022 (27)	1) Coercion/Consent; 2) Overpromising	1) Care Ethics	1) Regulation; 2) More research; 3) Medical oversight	Negative/Critical	1) Medical
thestar.co m	Kevin Jiang	Neuralink, Elon Musk's secretive brain chip company, could begin human trials in 6 months	2022 (28)	1) Animal welfare) 2) Safety	1) Care Ethics	1) More research	Balanced/Neutral	1) Medical; 2) Personal
The Hollywood Reporter	Sheri Linden	'Theater of Thought' Review: Werner Herzog Crafts a Bracing Exploration of Neurotechnology and Consciousness	2022 (30)	1) Autonomy; 2) Privacy; 3) Personhood/Identit y; 4) Exploitation	1) Care Ethics; 2) Categorical Imperative	1) Avoiding undesired results; 2) Regulation; 3) More research	Balanced/Neutral	1) Medical; 2) Personal

Targeted News Service	Editorial Staff	University of Rhode Island Professor Besio Issues Public Comment on Bureau of Industry & Security Proposed Rule	2022 (31)	1) Autonomy; 2) Coercion/Consent; 3) Exploitation; 4) Regulation	1) Utilitarianism	1) Avoiding undesired results; 2) More research; 3) Medical oversight	Balanced/Neutral	1) Medical; 2) Personal
Medtech Insight	Reed Miller	Activists Push US Government To Investigate Animal Abuse Charges Against Neuralink and UC Davis	2022 (32)	1) Safety; 2) Animal Welfare	1) Care Ethics; 2) Categorical Imperative	1) Avoiding undesired results; 2) More research; 3) Medical oversight	Negative/Critical	1) Medical
Irish Examiner	Ferris Jabar	BRAIN WAVE	2022 (33)	1) Safety; 2) Responsibility; 3) Personhood/Identity	1) Care Ethics; 2) Categorical Imperative	1) More research; 2) Medical oversight	Balanced/Neutral	1) Medical
Taranaki Daily News, New Zealand	Editorial Staff	Musk's rage against the machines	2022 (34)	1) Safety; 2) Animal Welfare; 3) Overpromising	1) Care Ethics; 2) Categorical Imperative	1) Avoiding undesired results; 2) Regulation; 3) More research; 4) Medical oversight	Balanced/Neutral	1) Medical; 2) Personal
The New York Times	Jonathan Moens	Brain Implant Allows Fully Paralyzed Patient to Communicate	2022 (36)	1) Safety; 2) Overpromising	1) Care Ethics; 2) Categorical Imperative	1) More research; 2) Medical oversight	Balanced/Neutral	1) Medical
Newstex Blogs	Bob Ambrogi	Neurotechnologically Augmented Lawyers? Billable Units of Attention? New Study Sees Threats and Opportunities for Neurotechnology and the Law	2022 (40)	1) Responsibility; 2) Regulation	1) Categorical Imperative	1) Avoiding undesired results; 2) Regulation	Negative/Critical	1) Medical; 2) Professional

Targeted News Service	Editorial Staff	Kernel Issues Public Comment on Bureau of Industry & Security Proposed Rule	2022 (41)	1) Biohacking; 2) Privacy; 3) Weaponization	1) Utilitarianism	1) Avoiding undesired results; 2) Regulation; 3) More research	Balanced/Neutral	1) Medical, 2) Professional; 3) Military
USA Today Online	Bailey Schulz	Elon Musk's Neuralink to host a 'show and tell.' What we know about the brain implant startup.	2022 (44)	1) Animal Welfare	1) Care Ethics	1) More research	Balanced/Neutral	1) Medical; 2) Personal
Newstex Blogs	Maria Hornbek-Copenhagen	Software edits images via human 'mind control'	2022 (45)	1) Privacy	1) Categorical Imperative	1) More research	Balanced/Neutral	1) Medical; 2) Personal
CE Noticias Financieras English	Editorial Staff	"Having a sensor on your head will be de rigueur in 10 years, just like everyone now has a smartphone."	2022 (48)	1) Privacy; 2) Weaponization; 3) Personhood/Identity; 4) Inequality; 5) Regulation	1) Utilitarianism; 2) Categorical Imperative	1) Avoiding undesired results; 2) Regulation; 3) More research	Balanced/Neutral	1) Medical; 2) Personal
The New York Times	Editorial Staff	Brain Implants Have Begun to Restore Functions, but Advances Are Slow	2022 (50)	1) Weaponization; 2) Animal Welfare	1) Care Ethics; 2) Categorical Imperative	1) Avoiding undesired results; 2) Regulation; 3) More research; 4) Medical oversight; 5) Not for public use	Balanced/Neutral	1) Medical; 2) Personal
Targeted News Service	Editorial Staff	University of Pittsburgh Issues Public Comment on Bureau of Industry & Security Proposed Rule	2022 (55)	1) Biohacking; 2) Autonomy; 3) Privacy; 4) Weaponization; 5) Privacy; 6) Responsibility	1) Utilitarianism	1) Avoiding undesired results; 2) More research	Balanced/Neutral	1) Medical; 2) Military
Newstex Blogs	Editorial Staff	Internet of Thinks: Securing the Brain Computer Interface (BCI)	2022 (56)	1) Biohacking; 2) Privacy; 3) Safety; 4)	1) Care Ethics; 2) Categorical Imperative	1) Avoiding undesired results; 2) Regulation; 3) More research; 4) Maintaining autonomy	Balanced/Neutral	1) Medical; 2) Personal

				Personhood/Identity				
Activist Post	Maryam Henein	Charles Lieber's Legal Escapades & Freedom Of The Mind	2022 (57)	1) Biohacking; 2) Autonomy; 3) Coercion/Consent; 4) Privacy; 5) Safety; 6) Animal Welfare	1) Categorical Imperative	1) Avoiding undesired results; 2) Regulation; 3) Maintaining choice; 4) Maintaining autonomy; 5) Not for public use	Negative/Critical	1) Medical; 2) Personal
Medical Xpress	Japan Science and Technology Agency	Survey of researchers and the public on attitudes toward BRAIN-AI convergence	2023 (1)	1) Privacy; 2) Responsibility; 3) Accessibility; 4) Inequality	1) Utilitarianism; 2) Care Ethics	1) Encouraging public involvement; 2) Avoiding undesired results; 3) Regulation; 4) More research	Balanced/Neutral	1) Medical; 2) Personal
ENP Newswire	Matt Shipman	The Future Is Now: Wrestling with Ethics, Policy and Brain-Computer Interfaces	2023 (2)	1) Autonomy; 2) Coercion/Consent; 3) Privacy; 4) Safety; 5) Animal Welfare; 6) Responsibility; 7) Personhood/Identity; 8) Inequality; 9) Regulation	1) Utilitarianism; 2) Care Ethics; 3) Categorical Imperative	1) Regulation; 2) More research	Balanced/Neutral	1) Medical; 2) Personal
MINT, New Dehli	Editorial Staff	Someone in my head	2023 (3)	1) Biohacking; 2) Autonomy	1) Categorical Imperative	1) Encouraging public involvement 2) Avoiding undesired results; 3) More research; 4) Maintaining autonomy	Balanced/Neutral	1) Medical
Cryptopolitan	John Palmer	Elon Musk's Ambitious Neuralink: Merging Humans with AI	2023 (4)	1) Privacy; 2) Safety	1) Categorical Imperative	1) Encouraging public involvement 2) Avoiding undesired results; 3) Regulation	Balanced/Neutral	1) Medical; 2) Personal

Alive	Sayani Chakraborty	NeuroStrike: Unleashing Warfare On Mind, Urgent Defenses Needed	2023 (5)	1) Autonomy; 2) Coercion/Consent ; 3) Privacy; 4) Weaponization	1) Care Ethics; 2) Categorical Imperative	1) Encouraging public involvement 2) Avoiding undesired results; 3) Regulation; 4) Medical oversight; 5) Maintaining choice; 6) Maintaining autonomy	Negative/Critical	1) Military

ReadWrite	Deanna Ritchie	Brain-computer interface volunteers wanted. Is the potential help worth it?	2023 (7)	1) Coercion/Consent ; 2) Safety; 3) Regulation	1) Utilitarianism	1) Regulation; 2) More research; 3) Medical oversight; 4) Maintaining choice	Balanced/Neutral	1) Medical; 2) Personal
Chiang Rai Times, Pakistan	Editorial Staff	Elon Musk's Neuralink Secures Approval For Human Trial In Paralysis Patients	2023 (8)	1) Privacy; 2) Safety; 3) Animal Welfare;	1) Care Ethics; 2) Categorical Imperative	1) Avoiding undesired results; 2) More research; 3) Medical oversight	Balanced/Neutral	1) Medical
CE Noticias Financieras English	Editorial Staff	Brain implants: a revolution in medicine	2023 (9)	1) Autonomy; 2) Privacy; 3) Safety	1) Care Ethics; 2) Categorical Imperative	1) Avoiding undesired results; 2) Regulation; 3) More research; 4) Medical oversight; 5) Maintaining autonomy; 6) Not for public use	Balanced/Neutral	1) Medical
TRT World	Editorial Staff	Elon Musk's Neuralink dilemma: Decoding minds, challenging ethics	2023 (10)	1) Autonomy; 2) Privacy; 3) Weaponization; 4) Safety; 5) Animal Welfare	1) Utilitarianism; 2) Categorical Imperative	1) Avoiding undesired results; 2) Regulation; 3) More research	Negative/Critical	1) Medical; 2) Personal; 3) Military

The Independent, UK	Anthony Cuthbertson	Elon Musk says monkeys implanted with Neuralink brain chips were ‘close to death’	2023 (12)	1) Safety	1) Care Ethics	1) Avoiding undesired results; 2) More research; 3) Medical oversight	Negative/Critical	1) Medical; 2) Personal
Express Computer	Peter McConville	How AI and Technology is Blurring the Lines Between Humans and Machines	2023 (14)	1) Privacy; 2) Personhood/Identity; 3) Accessibility; 4) Inequality	Other	1) Avoiding undesired results	Positive/Enthusiastic	1) Professional
Daily Star Online	Leigh Mcmanus	Elon Musk's Neuralink raises moral question about interference in 'God’s design'	2023 (17)	1) Safety; 2) Animal Welfare	Other	1) Avoiding undesired results; 2) Medical oversight; 3) Not for public use	Negative/Critical	1) Medical
TimesTech	Tanya Rashmi	Brain-Computer Interface: Pioneering the Future of Computing	2023 (18)	1) Coercion/Consent; 2) Privacy; 3) Safety; 4) Personhood/Identity	1) Utilitarianism; 2) Care Ethics	1) Regulation; 2) More research; 3) Maintaining choice	Positive/Enthusiastic	1) Medical; 2) Personal; 3) Professional
News Release Wire	Dr. Patricia Farrell	Brain-Computer Interfaces: Good or Evil Intentions Are Possible	2023 (19)	1) Biohacking; 2) Autonomy; 3) Coercion/Consent; 4) Privacy; 5) Weaponization; 6) Safety; 7) Personhood/Identity	1) Utilitarianism	1) Encouraging public involvement 2) Avoiding undesired results; 3) Regulation; 4) More research; 5) Maintaining choice; 6) Maintaining autonomy	Negative/Critical	1) Medical

The Independent, UK	Vishwam Sankaran	Elon Musk's Neuralink brain chip company says it now has FDA approval for human testing	2023 (22)	1) Safety; 2) Animal Welfare	1) Care Ethics	1) Avoiding undesired results; 2) More research; 3) Medical oversight	Negative/Critical	1) Medical
Voice & Data	Shubhendu Parth	Set ground rules for brain-to-device communications	2023 (26)	1) Coercion/Consent; 2) Privacy; 3) Safety	1) Care Ethics; 2) Categorical Imperative	1) Regulation; 2) More research; 3) Medical oversight	Balanced/Neutral	1) Medical
Zero Hedge	Tyler Durden	Experts Predict Mind-Controlled Devices May Be Common By 2040s	2023 (27)	1) Privacy; 2) Weaponization; 3) Responsibility; 4) Personhood/Identity; 5) Regulation	1) Care Ethics; 2) Categorical Imperative	1) Regulation	Balanced/Neutral	1) Medical; 2) Personal
Global Times, China	Leng Shumei	Brain-computer interfaces technology renews hope for diseases treatment, has a long way to go before mass application	2023 (31)	1) Autonomy; 2) Personhood/Identity; 3) Regulation	1) Care Ethics; 2) Categorical Imperative	1) Regulation; 2) More research; 3) Medical oversight; 4) Not for public use	Balanced/Neutral	1) Medical
The Guardian, London	Maanvi Singh	Elon Musk's Neuralink approved to recruit humans for brain-implant trial	2023 (32)	1) Safety; 2) Animal Welfare	1) Categorical Imperative	1) Regulation; 2) More research	Negative/Critical	1) Medical
TechCrunch	Devin Coldewey	Neuralink human testing has reportedly received one FDA rejection already	2023 (33)	1) Safety; 2) Animal Welfare	1) Care Ethics	1) Avoiding undesired results; 2) Regulation; 3) More research; 4) Medical oversight	Negative/Critical	1) Medical

MailOnline	Sam Tonkin	Would YOU sign up? Elon Musk's Neuralink is recruiting participants to trial its controversial brain implants	2023 (36)	1) Safety; 2) Animal Welfare	1) Care Ethics	1) Regulation; 2) More research	Negative/Critical	1) Medical; 2) Personal
CE Noticias Financieras English	Editorial Staff	Real cyborgs: Elon Musk's company Neuralink wants to merge minds and computers	2023 (39)	1) Animal Welfare; 2) Personhood/Identity	1) Care Ethics; 2) Categorical Imperative	1) Regulation; 2) Medical oversight	Negative/Critical	1) Medical; 2) Personal
FierceBioTech	Andrea Park	Neuralink's animal research oversight board may pose conflicts of interest: Reuters	2023 (42)	1) Safety; 2) Animal Welfare	1) Care Ethics	1) Avoiding undesired results; 2) Regulation; 3) More research; 4) Medical oversight	Negative/Critical	1) Medical; 2) Personal
USA Today	Mike Snider	Elon Musk's Neuralink has FDA approval to put chips in humans' brains. Here's what's next.	2023 (46)	1) Privacy; 2) Animal Welfare; 3) Overpromising	1) Care Ethics	1) Regulation; 2) More research; 3) Medical oversight	Balanced/Neutral	1) Medical; 2) Personal
Gizmodo	Nikki Main	Elon Musk's Neuralink Seeks Volunteers for Brain Chip Implant Study	2023 (48)	1) Safety; 2) Animal Welfare	1) Care Ethics; 2) Categorical Imperative	1) More research; 2) Medical oversight	Negative/Critical	1) Medical; 2) Personal
BruDirect	Editorial Staff	Mind over matter: Mind-controlled 'smart brain' devices may become common by 2040, says expert	2023 (49)	1) Autonomy; 2) Privacy; 3) Weaponization; 4) Responsibility	1) Utilitarianism; 2) Care Ethics	1) Avoiding undesired results; 2) More research; 3) Medical oversight4) Maintaining autonomy	Balanced/Neutral	1) Medical; 2) Personal; 3) Military
Business Standard	Devangshu Datta	What makes Elon Musk's brain implant devices a double-edged sword	2023 (54)	1) Biohacking; 2) Coercion/Consent; 3) Privacy; 4)	1) Care Ethics	1) Avoiding undesired results; 2) More research; 3) Maintaining choice	Negative/Critical	1) Medical; 2) Personal

				Animal Welfare				
The Daily Upside	Editorial Staff	Neuralink gets a look inside your head	2023 (64)	1) Safety	1) Care Ethics	1) More research; 2) Medical oversight	Balanced/Neutral	1) Medical
Times of Oman	DW	Can Elon Musk's Neuralink tech really read your mind	2023 (66)	1) Privacy; 2) Animal Welfare; 3) Inequality	1) Care Ethics	1) Avoiding undesired results; 2) More research; 3) Medical oversight	Positive/Enthusiastic	1) Medical; 2) Personal
Deutsche Presse-Agentur	Alice Lanzke	New brain scanner can read thoughts - or at least some of them	2023 (70)	1) Privacy; 2) Responsibility	1) Care Ethics	1) More research	Negative/Critical	1) Medical
MailOnline	Jonathan Chadwick	I became Britain's first CYBORG when I had a chip inserted in my arm 25 years ago - but it worries me when I hear the risks biohackers are taking today	2023 (71)	1) Biohacking; 2) Coercion/Consent; 3) Safety	Other	1) More research; 2) Medical oversight	Balanced/Neutral	1) Medical; 2) Personal
Daily Star Online	Reanna Smith	Fear-eradicating brain implants could allow soldiers to fly planes using their thoughts	2023 (75)	1) Coercion/Consent; 2) Weaponization	1) Categorical Imperative	1) Avoiding undesired results; 2) Maintaining choice	Balanced/Neutral	1) Medical; 2) Military

Nature	Liam Drew	The rise of brain-reading technology: what you need to know	2023 (76)	1) Autonomy; 2) Privacy; 3) Safety; 4) Overpromising; 5) Regulation	1) Care Ethics; 2) Categorical Imperative	1) Avoiding undesired results; 2) Regulation; 3) More research; 4) Maintaining autonomy	Balanced/Neutral	1) Medical; 2) Personal; 3) Professional
CE Noticias Financier as English	Editorial Staff	Elon Musk's brain implant company is looking for clinical trial candidates: "We're decades away from having any results"	2023 (78)	1) Safety; 2) Animal Welfare	1) Categorical Imperative	1) Avoiding undesired results; 2) More research; 3) Medical oversight	Negative/Critical	Medical
The Telegraph, UK	Matthew Field	The NHS surgeon challenging Musk's technology to connect your brain to the internet	2023 (81)	1) Safety; 2) Animal Welfare	1) Care Ethics	1) Avoiding undesired results; 2) More research; 3) Medical oversight	Balanced/Neutral	1) Medical; 2) Personal
The Conversation	Laura Y. Cabrera	New Neurotechnology Is Blurring The Lines Around Mental Privacy But Are New Human Rights The Answer?	2023 (90)	1) Autonomy; 2) Privacy; 3) Regulation	1) Categorical Imperative	1) Regulation; 2) More research; 3) Maintaining autonomy	Balanced/Neutral	1) Medical
Sunday Independent	Patrice Harrington	Face of the future?; Mind reader Closing the gap between human and machine has long been coming, but with 'neurotech', we're closer than ever. Earlier this month, Elon Musk's Neuralink received FDA approval to begin human trials, and thousands of patents have been filed for other uses. But some argue this is the beginning of the end of privacy...	2023 (91)	1) Privacy; 2) Inequality	1) Utilitarianism; 2) Care Ethics	1) Avoiding undesired results; 2) More research; 3) Medical oversight	Positive/Enthusia stic	1) Medical; 2) Personal; 3) Professional; 4) Military

The Conversation	Christina Maher	Our neurodata can reveal our most private selves. As brain implants become common, how will it be protected?	2023 (92)	1) Autonomy; 2) Privacy; 3) Weaponization; 4) Responsibility; 5) Regulation	1) Care Ethics; 2) Categorical Imperative	1) Avoiding undesired results; 2) Regulation; 3) More research; 4) Maintaining autonomy	Balanced/Neutral	1) Medical; 2) Military
The Independen t, UK	Steven Cutts	When sci-fi becomes reality: could brain-machine interfaces be right around the corner?	2023 (98)	1) Autonomy; 2) Animal Welfare; 3) Responsibility	1) Care Ethics; 2) Categorical Imperative	1) Avoiding undesired results; 2) Regulation; 3) More research; 4) Maintaining autonomy	Negative/Critical	1) Medical
MINT	Editorial Staff	The vast promise and harrowing pitfalls of neural implants	2023 (104)	1) Biohacking; 2) Autonomy; 3) Coercion/Consent	1) Care Ethics; 2) Categorical Imperative	1) Avoiding undesired results; 2) More research; 3) Maintaining choice; 4) Maintaining autonomy	Balanced/Neutral	1) Medical; 2) Personal
CE Noticias Financieras English	Editorial Staff	Brain-computer interfaces: a dystopian frontier for user experience	2023 (107)	1) Privacy	1) Categorical Imperative	1) Avoiding undesired results; 2) Regulation	Positive/Enthusia stic	1) Medical; 2) Personal; 3) Professional
Newstex Blogs	Howie Klein	Magadonians— The Polite Way Of Referring To MAGAts— Are Lining Up To Get Brains From Elon Musk	2023 (108)	1) Privacy; 2) Animal Welfare; 3) Overpromising; 4) Regulation	1) Categorical Imperative	1) Avoiding undesired results; 2) Regulation; 3) More research; 4) Not for public use	Negative/Critical	1) Medical; 2) Personal
Conservati ve Daily News	Editorial Staff	Jeff Bezos and Bill Gates Fund Brain Implant Company Synchron Competing With Elon Musk's Neuralink	2023 (116)	1) Biohacking; 2) Autonomy; 3) Privacy; 4) Safety; 5) Animal Welfare; 6) Regulation	1) Utilitarianism	1) Encouraging public involvement; 2) Avoiding undesired results; 3) Regulation; 4) More research; 5) Maintaining autonomy	Negative/Critical	1) Medical; 2) Personal

The Times, UK	Danny Fortson	What it’s like to have a mind-reading microchip in your brain	20123 (119)	1) Privacy; 2) Safety	1) Care Ethics; 2) Categorical Imperative	1) Avoiding undesired results; 2) More research; 3) Medical oversight	Positive/Enthusiastic	1) Medical; 2) Personal
The Australian	Natasha Robinson	BRAIN POWER	2023 (125)	1) Biohacking; 2) Privacy; 3) Regulation	1) Utilitarianism; 2) Care Ethics	1) More research; 2) Medical oversight	Positive/Enthusiastic	1) Medical
Global Data Point	Editorial Staff	Medical brain-computer interface technology advancing in China	2024 (4)	1) Privacy; 20 Safety	1) Care Ethics	1) More research; 2) Medical oversight; 3) Not for sale/profit	Balanced/Neutral	1) Medical
China Daily	Cheng Yu	Where free will, mental privacy inform protection of 'neuro rights'	2024 (11)	1) Biohacking; 2) Privacy; 3) Exploitation	1) Categorical Imperative	1) Regulation; 2) Not for sale/profit	Negative/Critical	1) Medical; 2) Personal
Opinion Nigeria	Abubakar Aliyu	Decoding the Future; The Promise and Perils of Neuralink's Human Testing	2024 (14)	1) Biohacking; 2) Privacy; 3) Safety	1) Care Ethics; 2) Categorical Imperative	1) Regulation; 2) More research	Negative/Critical	1) Medical; 2) Personal
CE Noticias Financieras English	Editorial Staff	The ethical dilemma of Elon Musk's chip	2024 (15)	1) Autonomy; 2) Privacy; 3) Personhood/Identity	1) Categorical Imperative	1) Regulation; 2) Maintaining autonomy	Negative/Critical	1) Medical; 2) Personal

Proactive Inventors, UK	Oliver Haill	Musk's Neuralink claims success with first brain computer implant	2024 (16)	1) Safety; 2) Animal Welfare	1) Care Ethics	1) Regulation	Negative/Critical	1) Medical
CE Noticias Financieras English	Editorial Staff	Elon Musk claims implanted brain chip is capable of moving a mouse with thought	2024 (18)	1) Privacy; 2) Exploitation	1) Categorical Imperative	1) Regulation	Negative/Critical	1) Medical
China Daily	Editorial Staff	Ethical guidelines set BCI research on right wavelength	2024 (39)	1) Overpromising; 2) Regulation	Other	1) Regulation	Balanced/Neutral	1) Medical
CE Noticias Financieras English	Editorial Staff	Musk announces implantation of first brain chip without clarifying purpose	2024 (41)	1) Safety	1) Care Ethics	1) More research	Balanced/Neutral	1) Medical
eHealth	Asawari Savant	Neuralink's Breakthrough: A Deep Dive into the Promise, Perils, and Ethical Dimensions of Brain-Computer Interfaces By Dr. Asawari Savant, eHealth Network - 03 February 2024	2024 (48)	1) Safety; 2) Responsibility	1) Categorical Imperative	1) Avoiding undesired results; 2) Regulation	Balanced/Neutral	1) Medical
Benzinga	Editorial Staff	Jeff Bezos, Bill Gates-Backed Synchron Set To Challenge Elon Musk's Neuralink With Upcoming Large-Scale Brain	2024 (49)	1) Safety; 2) Animal Welfare	1) Care Ethics	1) Regulation	Negative/Critical	1) Medical

		Implant Trial						
OneGreenPlanet	Trinity Sparke	Reports Reveal How Many Animals Were Sacrificed for Brain-Computer Science Before Neuralink!	2024 (50)	1) Animal Welfare	1) Categorical Imperative	1) Encouraging public involvement; 2) Regulation	Negative/Critical	1) Medical
HIT Consultant	Fred Pennic	First Human Receives Neuralink Brain Implant, Raising Hopes and Concerns	2024 (53)	1) Safety; 2) Overpromising	1) Care Ethics; 2) Categorical Imperative	1) More research	Negative/Critical	1) Medical
CE Noticias Financieras English	Editorial Staff	Neuralink, Elon Musk and the Pandora's Box of neurotechnologies	2024 (57)	1) Privacy; 2) Safety; 3) Responsibility; 4) Regulation	1) Categorical Imperative	1) Encouraging public involvement; 2) Regulation	Negative/Critical	1) Medical; 2) Personal; 3) Professional; 4) Military
Targeted News Service	Editorial Staff	Mass General Brigham: Implantable Brain-Computer Interface Collaborative Community to Drive Innovation in Neurotechnology	2024 (62)	1) Safety; 2) Accessibility	1) Categorical Imperative	1) Encouraging public involvement; 2) Regulation	Positive/Enthusiastic	1) Medical
CE Noticias Financieras English	Editorial Staff	Neuralink: Wellness or the first step towards "superhumans"?	2024 (67)	1) Biohacking; 2) Exploitation	1) Categorical Imperative	1) Regulation	Negative/Critical	1) Medical; 2) Personal
The Rising Nepal	Rastra Dhoj Karki	Neuralink: Bridging Gap Between Mind And Machine	2024 (70)	1) Autonomy; 2) Privacy; 3) Weaponization; 4) Safety	1) Care Ethics; 2) Categorical Imperative	1) Regulation	Balanced/Neutral	1) Medical

Mail & Guardian	Donrich Thaldar & Marietjie Botes	Mind matters: Navigating the ethical frontier of neurotechnology	2024 (71)	1) Biohacking; 2) Autonomy; 3) Privacy	1) Categorical Imperative	1) Regulation; 2) Maintaining autonomy	Negative/Critical	1) Medical; 2) Personal
The Daily Star	Ewan Gleadow	Fears Elon Musk's Neuralink could 'kill humans' as it's just 'invasive brain-computer'	2024 (72)	1) Safety	1) Care Ethics	1) Regulation	Negative/Critical	1) Medical
The Australian	Lorraine Finlay	Let's not elevate brain tech over our humanity	2024 (78)	1) Privacy	1) Categorical Imperative	1) Regulation; 2) Maintaining autonomy	Negative/Critical	1) Medical; 2) Personal
Yukon News	Omar H. Fares	The first Neuralink brain implant signals a new phase for human-computer interaction	2024 (90)	1) Autonomy; 2) Privacy; 3) Safety	1) Categorical Imperative	1) Regulation; 2) More research	Balanced/Neutral	1) Medical; 2) Personal
Thai News Service	Fred Schwaller	United States: Neuralink's telepathy brain chip: How weird is it?	2024 (94)	1) Privacy; 2) Safety	1) Categorical Imperative	1) Encouraging public involvement; 2) Regulation; 3) More research	Negative/Critical	1) Medical
The Peterborough Examiner	Lisa M. Krieger	What do we know about Neuralink?; Secrecy surrounding Musk's brain project and its human patient adds to controversy	2024 (118)	1) Safety; 2) Responsibility	1) Care Ethics	1) Medical oversight	Balanced/Neutral	1) Medical

Newstex Blogs	Wade Zhou	How technology is bridging brains with computers	2024 (127)	1) Safety; 2) Responsibility	1) Care Ethics	1) More research	Balanced/Neutral	1) Medical; 2) Personal
The Conversation	Laura Y. Cabrera	New Neurotechnology Is Blurring The Lines Around Mental Privacy – But Are New Human Rights The Answer?	2024 (134)	1) Privacy; 2) Personhood/Identity	1) Categorical Imperative	1) Encouraging public involvement; 2) Regulation; 3) Maintaining autonomy	Negative/Critical	1) Medical; 2) Personal
CE Noticias Financieras English	Editorial Staff	How Elon Musk's Neuralink brain implant 'expands the frontiers of the mind'	2024 (137)	1) Autonomy; 2) Responsibility; 3) Personhood/Identity	1) Categorical Imperative	1) Maintaining autonomy; 2) Not for sale/profit	Negative/Critical	1) Personal
Naked Capitalism	Yves Smith	Advances in Mind-Decoding Technologies Raise Hopes (and Worries)	2024 (141)	1) Privacy; 2) Exploitation	1) Care Ethics; 2) Categorical Imperative	1) Encouraging public involvement; 2) Regulation; 3) Maintaining autonomy	Negative/Critical	1) Medical; 2) Personal
Yerepouni Daily News	Editorial Staff	Several companies testing brain implants	2024 (148)	1) Privacy; 2) Safety	1) Care Ethics; 2) Categorical Imperative	1) Regulation; 2) More research; 3) Medical oversight	Negative/Critical	1) Personal; 2) Professional
The New Republic	Liz Jackson & Rua Williams	How Disabled People Get Exploited to Build the Technology of War	2024 (154)	1) Privacy; 2) Safety; 3) Exploitation	1) Care Ethics; 2) Categorical Imperative	1) Regulation; 2) Medical oversight; 3) Maintaining autonomy	Negative/Critical	1) Medical
